# Comment on “Development and Validation of Machine Learning‐Based Marker for Early Detection and Prognosis Stratification of Nonalcoholic Fatty Liver Disease”

**DOI:** 10.1002/advs.202515549

**Published:** 2025-09-23

**Authors:** Hongye Peng, Shaojie Duan

**Affiliations:** ^1^ Department of Traditional Chinese Medicine The Third Xiangya Hospital of Central South University 138 Tongzipo Road Changsha Hunan 410008 China; ^2^ Department of Geriatrics Taizhou Central Hospital (Taizhou University Hospital) Taizhou Zhejiang 318000 China

Recently, Lushan Xiao and colleagues^[^ ^1^ ^]^ developed a novel predictive model, the in silico scores for nonalcoholic fatty liver disease (NAFLD) (ISNLD), aimed at facilitating the early identification of individuals at high risk for NAFLD and predicting their progression to advanced liver disease and metabolic complications. The authors reported that ISNLD demonstrated robust discriminatory capacity for high‐risk NAFLD populations, with an area under the curve (AUC) exceeding 0.80, and significantly outperformed the widely used noninvasive index fibrosis‐4 (FIB‐4). Furthermore, in high‐risk individuals, ISNLD exhibited strong prognostic value for the subsequent development of severe liver diseases, including cirrhosis and hepatocellular carcinoma. These findings suggest that ISNLD represents a valuable tool for the early detection, individualized management, and targeted intervention of NAFLD, with considerable implications for both clinical practice and public health.

NAFLD is the most prevalent chronic liver disorder worldwide and can progress to cirrhosis or hepatocellular carcinoma, representing a significant threat to global health.^[^ ^2^ ^]^ Early diagnosis and timely intervention are essential to delay disease onset and progression, thereby improving population health outcomes. However, liver biopsy, which is the current diagnostic gold standard, has inherent limitations, including sampling variability, invasiveness, and high cost, which limit its feasibility for large‐scale clinical application.^[^ ^3^ ^]^ Consequently, there is an urgent need for efficient, noninvasive, and easily implementable diagnostic tools capable of accurately assessing prognostic risk.^[^ ^4^ ^]^ In this context, Lushan Xiao and colleagues^[^ ^1^ ^]^ developed the ISNLD, a model incorporating readily available clinical parameters and genetic single‐nucleotide polymorphisms (SNPs), thus circumventing the invasive nature of histological assessment. We consider ISNLD to hold considerable promise for early screening of NAFLD and for prognostic risk stratification in both clinical and public health settings.

After carefully reviewing the article, we have several questions we would like to discuss with the authors and other hepatology specialists. First, the authors compared the ISNLD model with the conventional FIB‐4 index to evaluate its potential advantages. However, FIB‐4 is primarily used to predict the risk of liver fibrosis,^[^ ^5^ ^]^ whereas indices such as the Fatty Liver Index,^[^ ^6^ ^]^ Hepatic Steatosis Index,^[^ ^7^ ^]^ and SteatoTest^[^ ^8^ ^]^ are widely used in clinical practice as classical tools for predicting NAFLD. Therefore, we would like to inquire whether the authors have directly compared ISNLD with these commonly used NAFLD prediction indices. In addition, beyond the AUC, accuracy, sensitivity, and specificity are also important metrics for assessing model performance. We are interested to know whether the authors have conducted comparative analyses of these metrics across the different models.

Second, the external validation dataset for the model was derived from a health examination cohort at Nanfang Hospital, in which the diagnosis of NAFLD was based solely on ultrasonography, given the absence of liver biopsy and MRI examinations. However, ultrasonography has limited sensitivity in detecting hepatic steatosis,^[^ ^9^ ^]^ particularly in cases of mild NAFLD, where there is a risk of underdiagnosis. We therefore wonder whether using ultrasonography as the reference standard for evaluating model performance might, to some extent, influence the accuracy of the results.

Third, this study used participants from the UK Biobank as the training and internal validation cohort, and a population from Nanfang Hospital in China as the external test set. Such a “UK‐to‐China” cross‐population design can, to some extent, evaluate the model's generalizability across populations with different genetic ancestries. However, given the substantial differences between the UK and Chinese populations in genetic background, allele frequencies, and linkage disequilibrium (LD) structures,^[^ ^10^ ^]^ the applicability and predictive accuracy of the model across distinct ancestral groups may be subject to certain limitations if adjustments to SNP weights or LD patterns are not undertaken. We would therefore be interested to learn whether any adjustments to SNP weights or LD patterns were undertaken to address these genetic differences between populations.

Finally, although the model demonstrates excellent predictive performance, it requires the acquisition of 10 clinical variables and information on 217 SNPs, which inevitably increases the complexity of data collection. It may therefore be worthwhile to further evaluate and reflect on the model's simplicity and its feasibility for large‐scale clinical application.

In summary, noninvasive diagnosis and risk assessment of NAFLD remain both a priority and a challenge in the medical field. We sincerely appreciate the authors’ outstanding work, which encompasses multiple dimensions—from clinical to genetic factors, and from NAFLD risk prediction to future outcome assessment. Nonetheless, there remain several aspects of ISNLD's application in NAFLD screening and diagnosis that merit further discussion. We hope that more hepatologists and liver disease specialists will take note of this innovative achievement, and we look forward to the early translation of ISNLD into clinical practice, with the aim of reducing the incidence of NAFLD and advanced liver disease.

## Conflict of Interest

The authors declare no conflict of interest.
